# Lycopene Modulates Oxidative Stress and Inflammation in Hypercholesterolemic Rats

**DOI:** 10.3390/ph15111420

**Published:** 2022-11-17

**Authors:** Tarfa Albrahim

**Affiliations:** Department of Health Sciences, Clinical Nutrition, College of Health and Rehabilitation Sciences, Princess Nourah bint Abdulrahman University, P.O. Box 84428, Riyadh 11671, Saudi Arabia; tarfa.ibrahim21@gmail.com

**Keywords:** lycopene, hypercholesterolemia, oxidative stress injury, inflammatory response, apoptosis, PPAR-γ

## Abstract

The complicated disorder of hypercholesterolemia has several underlying factors, including genetic and lifestyle factors. Low LDL cholesterol and elevated serum total cholesterol are its defining features. A carotenoid with antioxidant quality is lycopene. Examining lycopene activity in an animal model of hypercholesterolemia induced using food was the aim of this investigation. Triglycerides, LDL cholesterol, HDL cholesterol, and plasma total cholesterol were all measured. Biomarkers of renal and cardiac function were also examined. Apoptotic indicators, pro-inflammatory markers, and oxidative stress were also assessed. Additionally, the mRNA expression of paraoxonase 1 (PON-1), peroxisome proliferator-activated receptor gamma (PPAR-γ), and PPAR-γ coactivator 1 alpha (PGC-1α) in cardiac and renal tissues was examined. Rats showed elevated serum lipid levels, renal and cardiac dysfunction, significant oxidative stress, and pro-inflammatory and apoptotic markers at the end of the study. Treatment with lycopene significantly corrected and restored these changes. Additionally, lycopene markedly increased the mRNA expression of PGC-1α and PON-1, and decreased PPAR-γ expression. It was determined that lycopene has the capacity to modulate the PPAR-γ and PON-1 signaling pathway in order to preserve the cellular energy metabolism of the heart and kidney, which in turn reduces tissue inflammatory response and apoptosis. According to these findings, lycopene may be utilized as a medication to treat hypercholesterolemia. However, further studies should be conducted first to determine the appropriate dose and any adverse effects that may appear after lycopene usage in humans.

## 1. Introduction

Many nations struggle with the major issue of high cholesterol in blood. Since hypercholesterolemia is one of the main risk factors for the onset and progression of cardiovascular diseases, such as atherosclerosis and its complications, namely acute myocardial infarction, hypertension, accumulation of low-density lipoproteins (LDLs), stroke, and cerebral infarction, it is of great concern to medical professionals. Obesity and being overweight are associated with diet-related chronic diseases, such as type 2 diabetes, cardiovascular disease, various forms of cancer, and increased morbidity and mortality. Obesity typically results from an imbalance between energy input and energy production [1]. The level of low-density lipoproteins and other lipid-accumulating proteins rises when this ratio is out of balance, which causes adipose tissue to accumulate lipids. Although allopathic medications quickly cure cardiovascular irregularities and demonstrate the results, their severe side effects cannot be disregarded. As a result, the use of natural remedies to treat these chronic diseases is receiving more attention [2]. Furthermore, the nuclear receptor superfamily of ligand-activated transcription factors includes the peroxisome proliferator-activated receptor gamma (PPAR-γ) agonists which can treat type 2 diabetes and obesity-related insulin resistance [3]. Numerous studies have shown that PPAR-γ agonists have a wide range of actions that go beyond regulating lipid and glucose metabolism, such as anti-inflammatory characteristics [4,5,6].

According to the current theory, oxidative stress is the main mechanism through which dyslipidemia, particularly hypercholesterolemia, causes tissue damage or causes a number of disorders in humans. The oxidative modulation of LDL-c and glycoproteins, and glucose self-oxidation with enhanced formation of free radicals and lipid peroxidation products, are significant risk factors for ischemic heart disease, and have been found to be caused by hyperlipidemia and hypercholesterolemia. Increased aldehydes, including malondialdehyde (MDA) and conjugated aldehydes, have been linked to hyperlipidemia-induced free radical assault on membrane lipoproteins and polyunsaturated fatty acids, according to a number of studies [7]. A hydrolase enzyme known as paraoxonase 1 (PON-1) is strongly related to HDL in plasma. It is believed to have a preventive effect on the oxidation of low-density lipoproteins. Additionally, this enzyme plays a significant role in the antioxidant and anti-inflammatory properties of HDL-c. Additionally, LDL-c oxidative modification is prevented by PON-1. Systemic lipid peroxidation stress and potential cardiovascular risk are correlated with serum PON-1 activity. The molecular protective roles of naturally occurring antioxidants in biological systems have recently come under increasing scientific scrutiny. It has been suggested that antioxidants, such as flavonoids, polyphenols, vitamins C and E, and carotenoids shield the body’s system against reactive oxygen species. Due to their capacity to produce antioxidant effects, many efforts in the search for antioxidant chemicals are currently concentrated on diverse herbal plant extracts [8].

Lycopene is a primary dietary antioxidant that is produced and found in red and yellow fruits and plants. It is a member of the carotenoid family. It is primarily found in asparagus, (red) gac fruit, carrots, watermelons, papayas, and (yellow) parsley. Lycopene, which has the molecular formula C_40_H_56_, is a tetra-terpene lipophilic with conjugated double bonds (red colour factor) and an all-trans isomeric structure in plants. Lycopene was found by Di Mascio et al. in 1989 to be the most potent singlet-oxygen quencher among more than 600 naturally occurring carotenoids and to effectively slow the growth of reactive oxygen species (ROS) [9]. Lycopene’s pharmacological effects have been demonstrated as a natural or synthetic component of both conventional and novel medications. Lycopene has been shown in numerous studies to have anti-inflammatory and chronic disease-reducing properties [10,11,12]. Heart disease and cancer can be efficiently prevented and treated using lycopene. Lung and prostate cancer risks are greatly reduced as a result of the level of lycopene in serum and tissue. Lycopene’s anti-inflammatory, antioxidant, and anti-proliferative properties can prevent or treat heart failure, neoplasms, and cancer. Lycopene has been proven to have therapeutic and preventive effects by preventing oxidative stress, neuronal apoptosis, and inflammation, and by restoring mitochondrial activity in conditions or disorders of the central nervous system [13]. This study’s goals were to investigate the potential mechanism of lycopene’s anti-hypercholesterolemic effects on kidney and heart tissues that had been subjected to high-cholesterol diet-induced oxidative stress, inflammation, and apoptosis.

## 2. Results

### 2.1. Effect of Lycopene on Body Weight, Organ Weight, Glycemic Features, and Kidney Function Parameters

Feeding with a diet containing high cholesterol levels changed the body weight of rats non-significantly. However, lycopene administration to rats fed on HCD caused a non-marked decrease in the body weight of rats (Table 1). Furthermore, HCD feeding caused a significant increase (*p* < 0.05) in the absolute weights of the liver, kidney, and heart, and decreased significantly (*p* < 0.05) the relative weight of the liver and kidney. The changes in absolute and relative weights of vital organs were decreased significantly as a result of treating rats with lycopene compared with HCD-fed rats. Serum glucose levels in rats given HCD were clearly elevated (*p* < 0.05) compared to animals given a regular diet. However, lycopene administered to hypercholesterolemia rats showed a significant reduction (*p* < 0.05) in fasting glucose levels. However, compared to HCD-fed rats, rats that received a statin medication (AV) showed reduced glucose levels, although this decrease was not significant (Table 2). In comparison to controls, HCD rats had two times the amount of insulin, and after receiving lycopene, their plasma insulin levels were dramatically dropped. When compared to rats fed a regular diet, rats fed with HCD showed appreciable increases (*p* < 0.05) in serum levels of urea and creatinine. However, lycopene or AV treatment in rats caused significant drops (*p* < 0.05) in the serum levels of renal function indicators compared to the HCD-supplemented group (Table 2).

### 2.2. Effect of Lycopene on Lipid Profile

**Figure 1:**
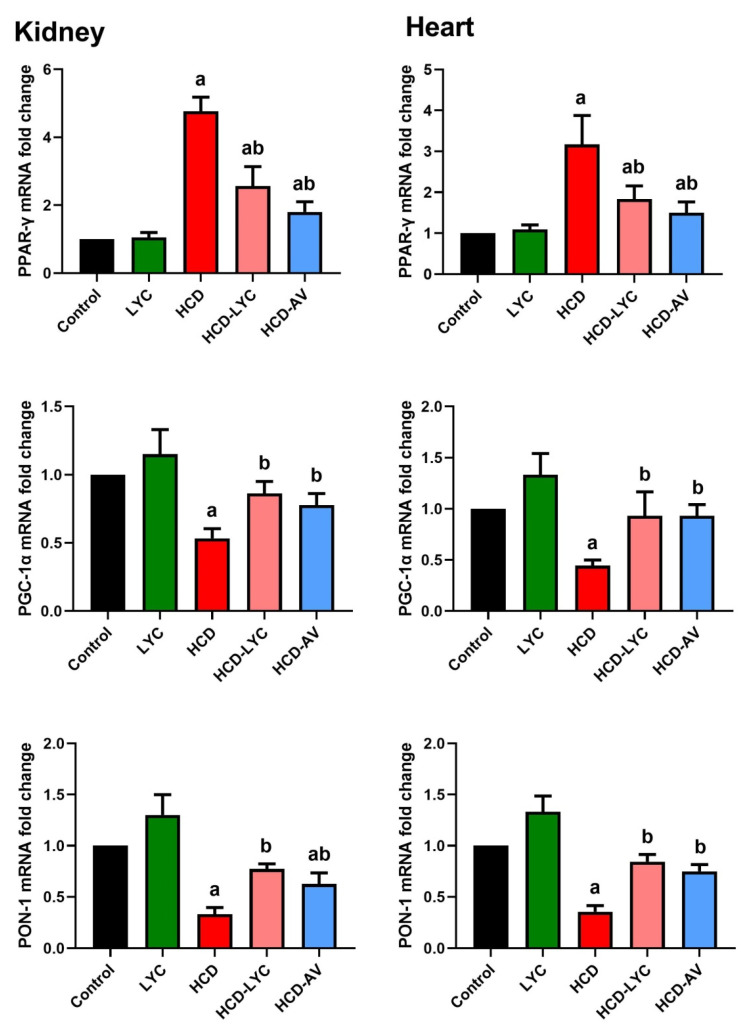
Effects of dietary cholesterol and lycopene on mRNA expression of peroxisome proliferator-activated receptor gamma (PPAR-γ), PPAR-γ coactivator 1 alpha (PGC-1α), and paraoxonase 1 (PON-1) in cardiac and renal tissues in rats over 12 weeks. Letters a and b indicate statistically significant differences between control and HCD groups, respectively, at *p* < 0.05.

When compared to rats given a regular diet, rats fed a HCD showed appreciable increases (*p* < 0.05) in TG, TC, LDL-c, and vLDL-c levels along with statistically significant decreases in HDL-c levels. However, the hypercholesterolemic group that received lycopene or AV showed significant reductions (*p* < 0.05) in TG, TC, and LDL-c as well as increases in HDL-c levels (Table 2). Rats subjected to HCD showed an atherogenic index that was significantly higher (*p* < 0.05) than rats fed a conventional diet (Table 2). As opposed to rats fed HCD, hypercholesterolemic rats given lycopene experienced a significant (*p* < 0.05) reduction in the AL. As anticipated, AV administration markedly reduced AL in comparison to rats that were fed HCD or were the control group. In hypercholesterolemic rats, both with and without lycopene treatment, cardiac and renal PPAR-γ and PGC-1α were evaluated. The PPAR-γ expression was significantly upregulated (*p* < 0.05) and PGC-1α expression was significantly downregulated (*p* < 0.05) in the HCD group compared to the control group of rats. However, downregulation (*p* < 0.05) of PPAR-γ and upregulation (*p* < 0.05) of PGC-1α expression in cardiac and renal tissues after lycopene treatment was observed, which demonstrated a strong anti-diabetic and anti-adipogenesis impact and a cellular energy metabolism modulation produced by lycopene administration (Figure 1). Moreover, the present study was extended to determine PON-1 expression in the kidney and heart tissues. Here, PON-1, the antioxidant enzyme of HDL-c, determines the proper function of HDL-c. Indeed, PON-1 expression was higher in HCD-LYC and HCD-AV than in HCD (Figure 1), which could account for the lower plasma cholesterol levels.

### 2.3. Effect of Lycopene on Cardiac Markers Following Hypercholesterolemia in Rats

**Figure 2:**
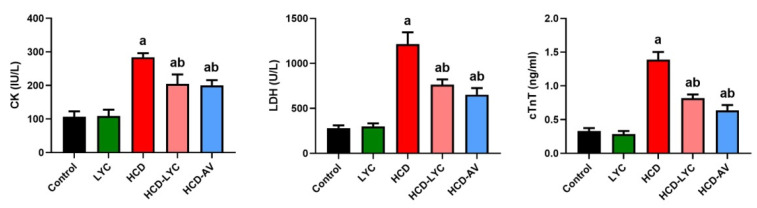
Effects of dietary cholesterol and lycopene on serum creatine kinase MB (CK), lactate dehydrogenase (LDH), and cardiac troponin T (cTnT) in rats over 12 weeks. Letters a and b indicate statistically significant differences between control and HCD groups, respectively, at *p* < 0.05.

In contrast to their controls, feeding with HCD elicited significant increases (*p* < 0.05) in the serum levels of CK, LDH, and cTnT (Figure 2). However, giving lycopene or AV to rats who had received HCD significantly reduced (*p* < 0.05) the increased CK, LDH, and cTnT compared to the model group.

### 2.4. Effect of Lycopene on Redox Homeostasis Following Hypercholesterolemia in Rats

**Figure 3:**
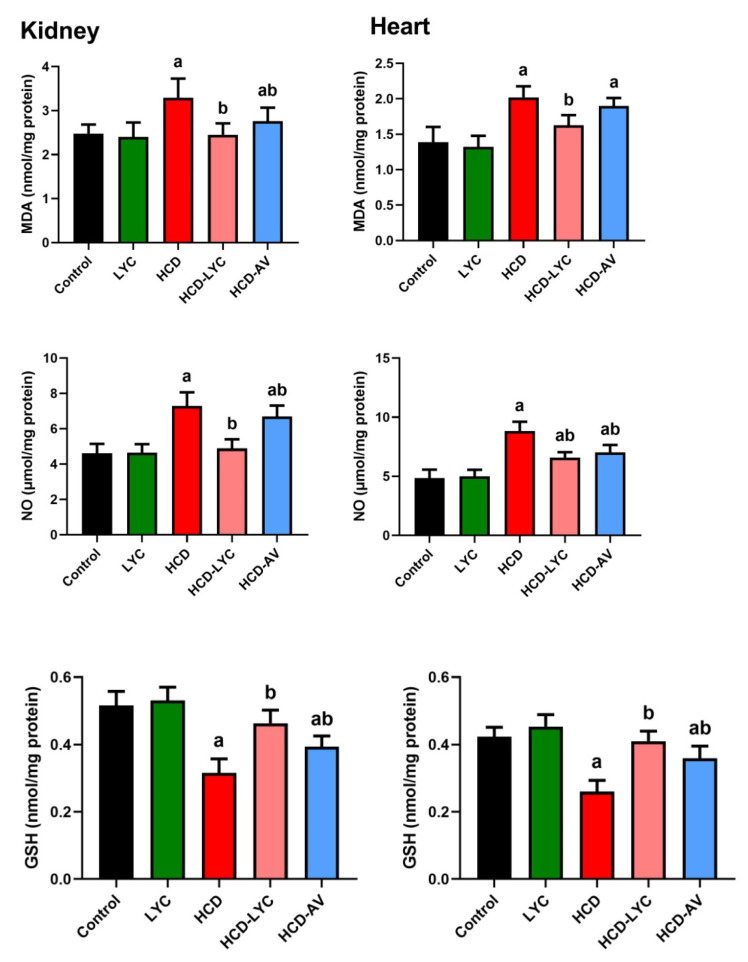
Effects of dietary cholesterol and lycopene on cardiac and renal malondialdehyde (MDA), nitric oxide (NO), and glutathione (GSH) levels in rat over 12 weeks. Letters a and b indicate statistically significant differences between control and HCD groups, respectively, at *p* < 0.05.

**Figure 4:**
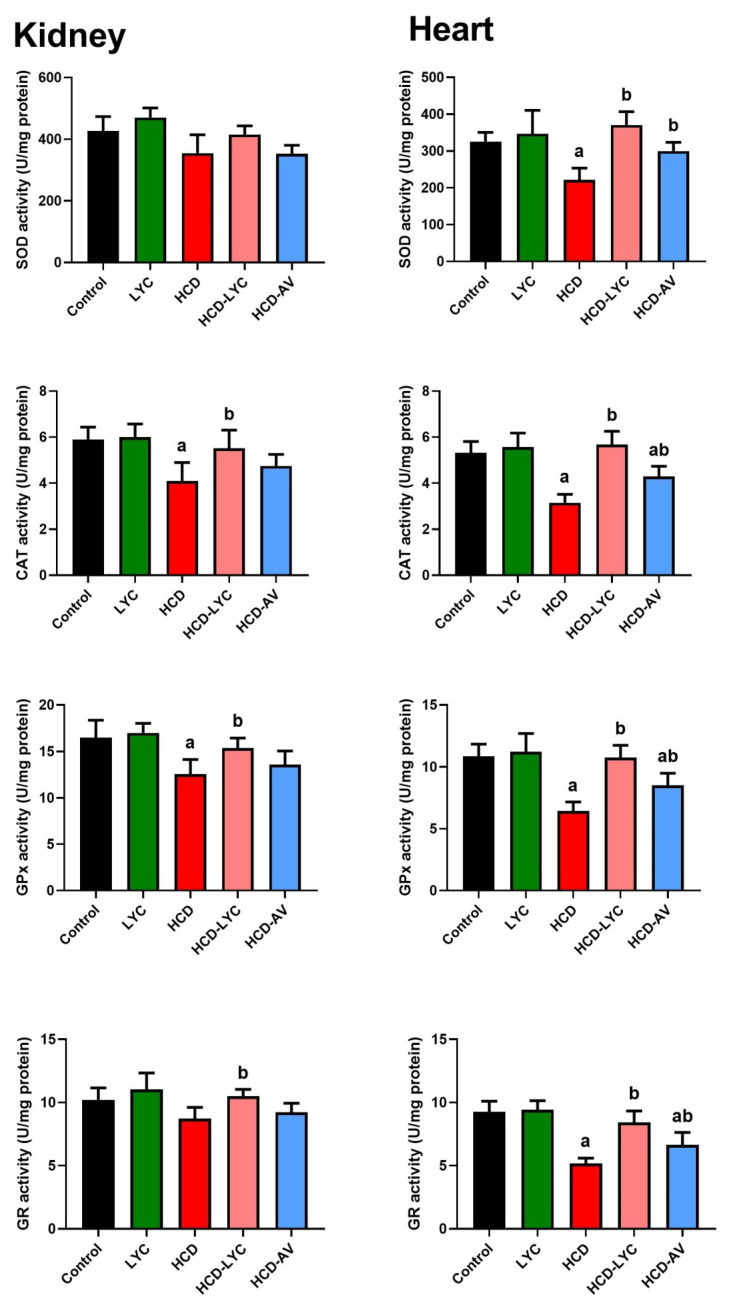
Effects of dietary cholesterol and lycopene on cardiac and hepatic superoxide dismutase (SOD), catalase (CAT), glutathione reductase (GR), and glutathione peroxidase (GPx) activities in rats over 12 weeks. Letters a and b indicate statistically significant differences between control and HCD groups, respectively, at *p* < 0.05.

The oxidative effect of HCD on cardiac and renal tissues was evident in the increased (*p* < 0.05) MDA and NO generation in HCD-challenged rats compared to controls. The endogenous antioxidants GSH, SOD, CAT, GPx, and GR showed a significant depletion (*p* < 0.05) after ingesting HCD. Expectedly, significant redox status improvement was observed after lycopene administration, as evidenced by the decreased pro-oxidants (MDA and NO) and increased cellular antioxidants (GSH, SOD, CAT, GPx, and GR) in comparison to rats fed HCD (Figure 3 and Figure 4).

### 2.5. Effect of lycopene on Inflammatory Response Following Hypercholesterolemia in Rats

**Figure 5:**
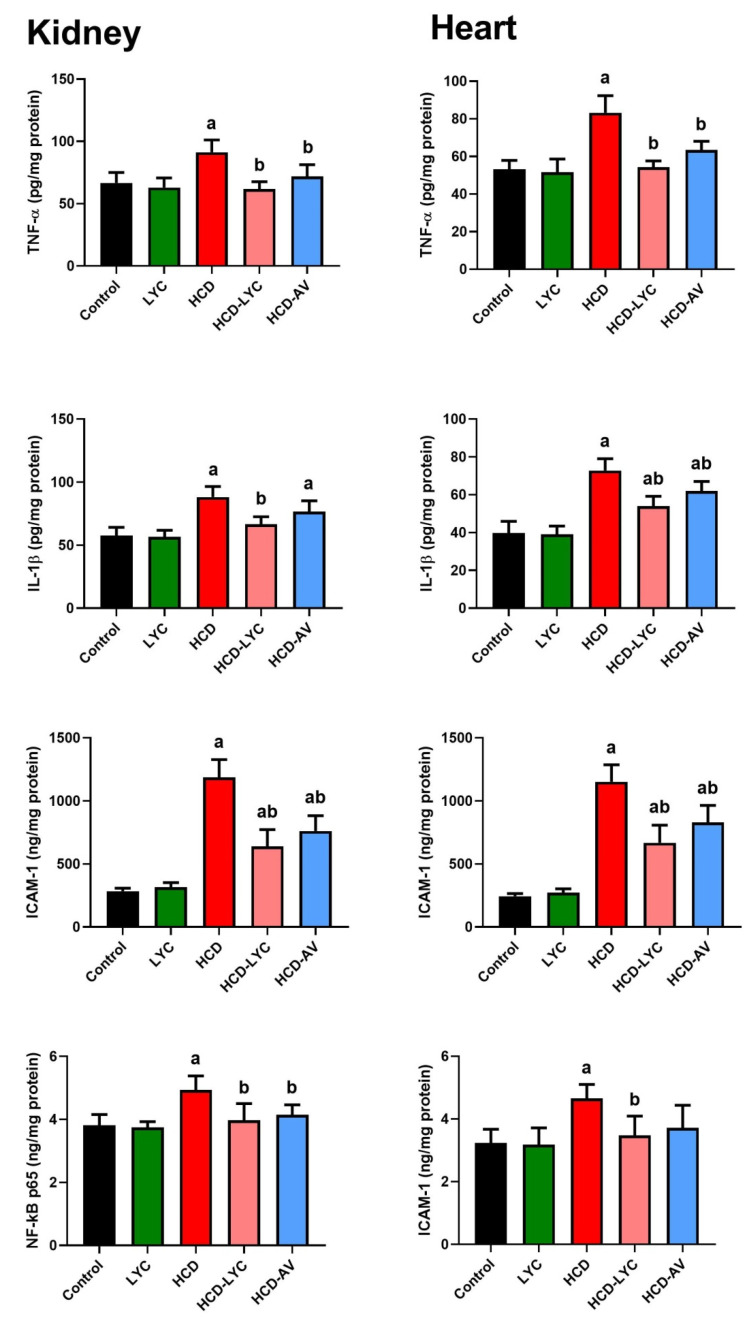
Effects of dietary cholesterol and lycopene on cardiac and renal tumor necrosis factor-alpha (TNF-α), interleukin-1 beta (IL-1β), nuclear factor kappa B (NF-κB), and intercellular adhesion molecule 1 (ICAM-1) levels in rats over 12 weeks. Letters a and b indicate statistically significant differences between control and HCD groups, respectively, at *p* < 0.05.

The levels of TNF-α and IL-6, as well as its activator, NF-κB, were markedly elevated (*p* < 0.05) in HCD-fed rats compared to controls, as shown in Figure 5. However, compared to rats fed HCD, animals treated with lycopene or AV displayed a significant decline in all inflammatory markers. These results show that lycopene has an anti-inflammatory impact on the HCD-induced inflammatory response linked to the development of hypercholesterolemia. Since hyperlipidemia is a major risk factor for atherosclerosis, atherogenic-related proteins were also analyzed. Leukocytes and platelets play important roles in atherosclerosis progression. Furthermore, adhesion molecules, such as intercellular adhesion molecule-1 (ICAM-1), assist monocyte migration to sites of inflammation. Monocytes bound to ICAM-1 and differentiated into macrophages. Hence, the present study was extended to determine ICAM-1 levels in heart and kidney tissue. The ICAM-1 was hardly noticeable in the hearts and kidneys of control animals, whereas HCD substantially enhanced its expression. Here, ICAM-1 is the endothelium ligand for the neutrophil receptor CD11b/CD18 (Figure 5). Lycopene and AV administration significantly decreased the rise in ICAM-1 expression brought on by the HCF diet (*p* < 0.05).

### 2.6. Effect of Lycopene on Apoptotic Markers Following Hypercholesterolemia in Rats

**Figure 6:**
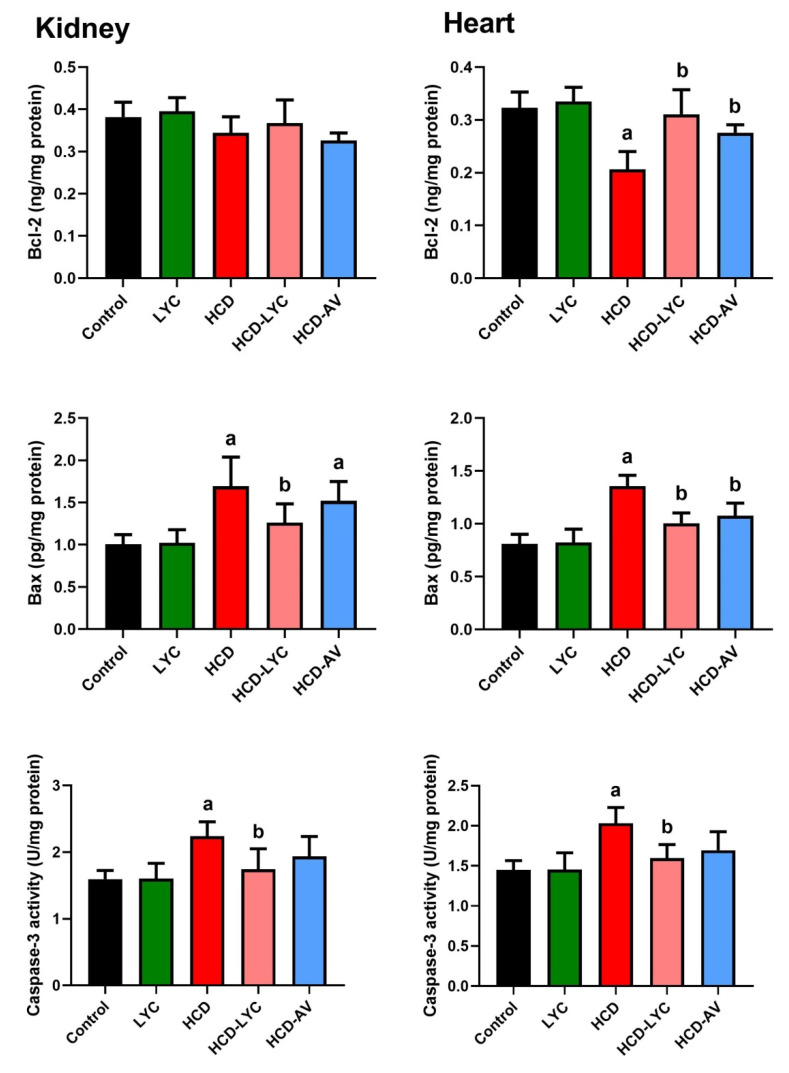
Effects of dietary cholesterol and lycopene on cardiac and renal B-cell lymphoma 2 (Bcl-2), BCL2 associated X, apoptosis regulator (Bax), and caspase-3 levels in rats over 12 weeks. Letters a and b indicate statistically significant differences between control and HCD groups, respectively, at *p* < 0.05.

In contrast to the control group, Figure 6 demonstrates that the HCD-fed rats exhibited clearly raised levels of Bax and caspase-3 (*p* < 0.05) as well as clearly decreased levels of Bcl-2 (*p* < 0.05) in cardiac and renal tissues. In contrast, lycopene or AV administration to hypercholesteremic rats reduced the levels of Bax and caspase-3 in both organs noticeably (*p* < 0.05) and increased the levels of Bcl-2 in comparison to the model group. Between the control and lycopene-treated groups, there were no discernible differences (*p* > 0.05) in the levels of either of the measured markers.

## 3. Discussion

Since various studies revealed the involvement of oxidative stress, inflammation, and apoptosis in the development and progression of atherosclerotic disease and its consequences, the use of antioxidant chemicals in the treatment of hypercholesterolemia has been rising [14,15]. Accordingly, the results of this study point to the protective role of lycopene in a hypercholesterolemia model, which is supported by the decline in plasma levels of the lipid profile, oxidative stress biomarkers, and pro-inflammatory cytokines, as well as the elevation of PGC-1 and PON-1 mRNA expression and the decrease in PPAR- expression.

It is well recognized that an increase in serum cholesterol can cause atherosclerosis, which impairs organ function by gradually decreasing blood flow to the organs. The improvement and occurrence of atherosclerosis and CAD are linked to reduced serum cholesterol levels, according to numerous studies [16,17]. Despite the fact that a number of actions are advised to treat hypercholesterolemia, modern lifestyles prevent people from doing so effectively. Current anti-hyperlipidemia medications, such as statins, block the HMG-CoA reductase enzyme, which produces cholesterol in the liver. The rate-limiting step in the synthesis of hepatic cholesterol is the enzymatic activity of 3-OH-3-methylglutarylcoenzymeA (HMG-CoA), which is competitively inhibited by atorvastatin. The HMG-CoA reductase is inhibited, which results in less cholesterol being produced in the liver and less LDL cholesterol being circulated in the blood [18]. Researchers are, therefore, interested in herbal medicines because they include antioxidants that may be a safe, effective, and affordable form of treatment [19]. According to the current investigation, eight weeks of HCD feeding improved both the serum lipid profile and AI. Treatment with lycopene reduced AI and a high serum lipid profile. This suggests that lycopene may be useful in reducing atherosclerosis, which eventually stops the development of CAD.

The principal organ in charge of preserving the balance of cholesterol is the liver. According to a number of lines of evidence, HCD increases the amount of hepatic cholesterol, which in turn increases the production of triglycerides [20]. In this study, HCD-induced rats had measurable increases in TC, TG, LDL-c, and vLDL-c cholesterol as well as a sizable drop in HDL-c. The TG-rich lipoproteins are often good indicators of the presence of cholesterol-rich particles that deposit cholesterol in the artery wall. Additionally, high LDL-c levels can result in the growth of “arterial atherosclerotic lesions”. The present study’s findings showed that lycopene supplementation considerably reduced hyperlipidemia, indicating that lycopene may be advantageous for the lipid and lipoprotein profile and that it might slow the development of atherosclerosis. The current study demonstrated that lycopene protects against altered lipid metabolism in HCD-fed rats.

As members of the nuclear hormone receptor superfamily, PPARs control a number of physiological processes, including cell differentiation, inflammation, glucose and lipid homeostasis, and inflammatory responses [21]. Triglyceride clearance, hepatic steatosis, and hyperlipidemia are all impacted by PPAR-α [22]. The PPARs-agonists (GW409544 and rosiglitazone) reduced hyperlipidemia via boosting malonyl-CoA decarboxylase protein production (MCD), which reduces the risk of myocardial infarction in patients with metabolic syndrome [23]. In obese, insulin-resistant monkeys, they have been observed to increase HDL cholesterol and decrease LDL cholesterol [24]. According to several studies, HCD treatment causes hepatic damage in the form of liver steatosis in rodents [21,25], and PPARs play a crucial role in modulating the effects of high-fat diets on liver steatosis [23]. The results of this investigation showed that lycopene decreased the levels of PPAR-α mRNA expression in the heart and kidney, resulting in cardiac and renal damage. As per the obtained data, a Coptidis rhizoma, Scutellariae radix, Rhei rhizoma, and Pruni cortex mixture is effective in preventing high-cholesterol diet-induced hyperlipidemia by regulating PPARs [21]. These findings suggested that lycopene reduces lipid buildup in hyperlipidemia and the renal and that cardiac damage brought on by HCD is caused by modulating the expression of PPARs.

By activating a number of genes involved in the tricarboxylic acid (TCA) cycle and antioxidant defense, PGC-1α is a crucial regulator of mitochondrial biogenesis. Additionally, PGC-1α promotes the mitochondrial transcription factor A (Tfam), a crucial component for the transcription, translation, and repair of mitochondrial DNA (mtDNA) [17,26]. The HCD reduced the expression of PGC-1α in this study. Improvements in cardiac and renal function have been linked to PGC-1α and mitochondrial function restoration [17]. Lipid buildup in cells impairs PGC-1α expression and mitochondrial performance, which promotes tissue dysfunction [17,26]. In the present investigation, HCD enhanced cholesterol buildup in cardiac and renal tissues, which was connected with a decrease in the levels of PGC-1α mRNA. The results of the current investigation showed that LYC reduced the HCD-induced decline in PGC-1α. The obtained results are consistent with the study of Wan et al. [27], who found that dietary LYC alleviated mitochondrial swelling, increased ATP levels, and upregulated the mRNA expression levels of PGC-1α in aflatoxin B1 challenged broilers. The authors concluded that LYC supplementation might alleviate mitochondrial dysfunction by improving mitochondrial biogenesis. Furthermore, Zhao et al. [28] found that LYC mitigated Di (2-ethylhexyl) phthalate-induced hepatic mitochondrial dysfunction in mice.

The current findings demonstrate a substantial rise in the cardiac markers CK, LDH, and cTnT in the serum of HCD rats, pointing to morphological and functional alterations in the heart muscle and indicating disruption of the integrity of the cell membrane. Due to the rupture and uncontrolled permeability of cell membranes, cardiac markers are released into the bloodstream from cardiomyocytes, indicating higher enzyme activity in the HCD group. Lycopene taken orally significantly decreased the CK, LDH, and cTnT in rats fed with a HCD. In HCD-induced rats compared to control rats, the preventive impact of lycopene reduces the severity of cardiac anomalies and may be responsible for the observed modifications. According to Ferreira-Santos et al. [29], lycopene has been linked to changed cardiac indicators, lipid peroxidation markers, and antioxidant status in rats with hypertension induced by Angiotensin II.

Hyperlipidemia and hypercholesterolemia are crucial in a number of kidney disease phases, including the development of obesity-related glomerulopathy and the advancement of metabolic syndrome and obesity [30]. High levels of lipid deposition in the kidney cause sizable modifications in renal subcellular structures (renal cortex) [15]. Large localized blood arteries enlarge due to excessive adiposity, and subcapsular adipocyte accumulation leads to glomerular atrophy and necrosis, which impairs the filtration of several molecules and raises their plasma levels. Due to a large consumption of saturated fatty acids, problems of the vascular system and nephropathy have also been described. One significant risk factor for the development of chronic renal disease (CKD) is hypercholesterolemia [30]. Additionally, renal disease advances faster when these anomalies are present. Such nephrotoxic and abnormal circumstances result in renal dysfunction as well as an imbalance in the amounts of electrolytes and small molecules inside and outside of the cells [12]. These are connected with increased oxidative stress and kidney cell death, which cause inflammation and compromise renal function. Consistent with earlier findings, the current study’s findings showed that feeding with HCD induces significant elevations in blood urea and creatinine, which are signs of compromised kidney function in rats receiving HCD [30]. The HCD causes an increase in protein catabolism, which causes these animals to produce more urea. Furthermore, in HCD-fed conditions, impaired renal function that resulted in decreased renal clearance may have made things worse. However, lycopene treatment restored blood urea and creatinine levels to normal in the LYC-treated group, offering a defense against HCD-induced kidney impairment. These protective properties of lycopene could be ascribed to both its antioxidant properties and its ameliorative activity against metabolic alterations observed in HCD-fed conditions. Similar renoprotective effects of lycopene on rising blood levels of uric acid and creatinine have been shown in rats. This is due to the direct role played by these natural therapeutic substances in the recovery of damaged organs and harmed cell lines [1].

In the current investigation, HCD led to an excessive buildup of MDA in the rat heart and kidney. Lipid peroxidation produces MDA. The equilibrium between antioxidants and oxidants in the rat’s organs would be upset during fatty acid oxidation due to the increased ROS generation. A vicious cycle results when ROS rises and antioxidant enzymes and GSH fall, exacerbating the effects of oxidative stress. Endothelial cells are harmed by hyperlipidemia, which results in simultaneous macrophage invasion and lipid deposition, important steps in the progression of atherosclerosis. Through two mechanisms which cause direct harm to cell membranes and nuclei and generation of oxidized lipoproteins, ROS production acts as a crucial mediator in hyperlipidemia. Antioxidant enzymes lower oxidant concentrations, which lowers lipid peroxidation. These recognized atherosclerosis-fighting enzymes include SOD, GPx, GR, CAT, and PON-1 [31]. In the study, lycopene-treated rats had considerably lower MDA levels. It was also discovered that the CAT, GR, GPx, and SOD enzyme activities were increasing. These results imply that oral lycopene therapy can prevent renal and cardiac damage caused by HCD in rats and lower the risk of atherosclerosis by lowering oxidative damage. Similar to the findings previously discussed, lycopene treatment modulates the redox status in plasma and kidney homogenates of rats with oxidative stress caused by cadmium and streptozotocin-induced diabetes [32], furan-induced hepatotoxic and hematologic changes in diabetic rats [33], Wistar rats fed a high-fat diet [34], and liver homogenates of rats with hypertension induced by Angiotensin II [29].

The HDL-linked enzyme paraoxonase 1 (PON-1) protects LDL-c and HDL-c against lipid peroxidation [35]. Furthermore, PON-1 has been shown to be a protective factor in disease associated with inflammation and oxidation, such as diabetes mellitus and non-alcoholic fatty liver diseases. Several studies demonstrate how polyphenols can induce PON-1. The preservation of the enzyme -SH group supports the idea that plasma redox state plays a significant role in controlling PON-1 activity. The current research demonstrates the antioxidant function of lycopene by showing that it can reverse the effects of hypercholesterolemic stress in rats given atherogenic supplements. The obtained data of the current study are in agreement with Kumar et al. [36], who found that hesperidin supplementation significantly upregulated the expression of PON-1 in hyperlipidemic rats.

In addition to having elevated serum lipid levels, hyperlipidemia is also an inflammatory condition, since excessive lipid buildup has been shown to set off localized inflammatory responses [21,37]. As seen in nonalcoholic steatohepatitis, the inflammatory processes primarily correspond to increased local fat accumulation [38]. Additionally, inflammatory processes take place throughout the course of cardiovascular illness [39,40]. It has been demonstrated that inhibiting inflammatory cytokines can lower the prevalence of cardiovascular disease [41]. According to the findings of this study, lycopene dramatically decreased the levels of inflammatory cytokines (TNF-α and IL-6), NF-κB, and adhesion molecules including ICAM-1 in rats receiving HCD. Numerous cytokines and mediators contributed to the production of plaque as atherosclerotic disease progressed [42]. Atherosclerotic plaques have been found to express IL-family members and their receptors. Additionally, in apolipoprotein E knockout mice, TNF-α suppression lowers atherosclerosis [43]. An atherogenic cytokine called IL-6, which is mostly released by smooth muscle cells (SMCs), promotes the production and release of atherothrombotic molecules, such as fibrinogen and plasminogen activator inhibitor-1. Additionally, it promotes the expression of adhesion molecules and chemokines on the surface of blood vessels, as well as the production and release of C-reactive protein from hepatocytes and cardiac tissues [44]. The regulation of genes involved in inflammation, degenerative changes, and growth control is thought to be influenced by NF-κB activation. By controlling the expression of cytokines and adhesion molecules including vascular cell adhesion molecule, ICAM-1, and platelet endothelial cell adhesion molecule-1, NF-κB may play this role. A transcription factor called NF-κB controls a variety of cellular processes, from inflammation to cell death [45]. As implied by the title “nuclear factor”, NF-κB must move from the cytoplasm to the nucleus in order to bind appropriate nuclear DNA sequences. These cytokines can induce further production of ROS and, hence, more cell and tissue damage [37]. In 2002, Liu et al. [46] demonstrated that NF-κB is expressed on platelets and that thrombin-induced platelet activation causes I-kappa B alpha (I-κB), an NF-κB inhibitor, to degrade. Accordingly, the collected data imply that lycopene inhibitory effects on inflammatory cytokines and mediator levels are connected with protection against the onset of cardiac and renal damage through inhibiting the NF-κB pathway.

The current findings also showed that hypercholesterolemia increased apoptosis in the heart and kidneys of rats. The findings showed a simultaneous rise in Bax and caspase-3 activation together with a decrease in Bcl-2 levels. Similar to this, Cheng et al. found that an 8 week diet high in cholesterol caused myocardial apoptosis in hamsters, which was accompanied by a rise in BID and Bax expression [47]. These findings are consistent with those of Zhu et al. who demonstrated that a 12 week hypercholesterolemic diet increased cardiac expression of Bax and decreased BCL-xL in swine, but had no effect on caspase-3 [48]. On the other hand, Osipov et al. demonstrated in a Yucatan swine model that hyperlipidemia did not worsen cardiac apoptosis after ischemia and reperfusion based on the findings of decreased cleaved PARP and no difference in activation of caspase-3 [49]. Other tissue types have also revealed hypercholesterolemia to affect apoptosis. For instance, Perales et al. found that vascular smooth muscle cells exposed to 25-hydroxycholesterol had increased Bax expression [50]. Additionally, it was discovered that hypercholesterolemia significantly increased the expression of proprotein convertase subtilisin/Kexin type 9 (PCSK9), BACE1, caspase-3, and Bax while only marginally increasing the expression of Bcl-2, suggesting that hyperlipidemia caused neuronal apoptosis in the hippocampus of apoE(/) mice via the Bcl-2/Bax–caspase-3 signaling pathway [51]. Additionally, Yao et al. [52] discovered that p53 is implicated in the pathophysiology of lipid-induced kidney injury and that lipid peroxidation is a crucial event that causes DNA damage. According to Othman et al. [37], HFD/STZ significantly increased Bax and caspase-3 while significantly decreasing Bcl-2 in the liver and kidney tissues, indicating apoptosis. Therefore, even if different models of hypercholesterolemia show distinct constellations of altered apoptotic markers, the majority of these studies show that there is a substantial correlation between enhanced cardiac and renal apoptosis and hypercholesterolemia.

In the current study, lycopene therapy significantly reduced cell apoptosis in hypercholesterolemic rats’ hearts and kidneys. Previous authors reported that lycopene has anti-apoptotic activity [10,53,54]. The mitochondrial membrane sustains significant damage as a result of oxidative stress, which then changes the ratio of proapoptotic (such as Bax) to antiapoptotic (such as Bcl-2) Bcl-2 family members. As a result, cytochrome c is released, the caspase cascade is activated, and DNA damage results [10]. Additionally, lycopene greatly decreased Bax levels and increased Bcl-2 levels in cardiac injury caused by myocardial ischemia [55]. The ability of this compound in modulating the extrinsic and intrinsic pathways of apoptosis to diminish the expression of caspase-9 produced by aristolochic acid in renal tissue was demonstrated after treatment with lycopene [56].

## 4. Materials and Methods

### 4.1. Animals and Experimental Design

Male Wistar rats, 8–10 weeks old, weighing 180–210 g, were housed in typical conditions with unrestricted access to food and water. Prior to use, every animal underwent a week of acclimation. The Princess Nourah Bint Abdulrahman University’s Institutional Animal Care and Use Committee (IACUC) authorized the methods, and all studies were carried out in compliance with generally accepted standards in a designated pathogen-free environment (Approval No. HAP-01-R-059; IRB Registration No. 21-0502; Category of Approval: EXEMPT; 23 December 2021). Rats were divided into five groups, with *n* = 7 in each group. The first group, designated as the control, had a regular basal diet up until the completion of the experiment. The rats in the second group (LYC), which received lycopene (Sigma Chemicals, St. Louis, MO, USA) at a dose of 50 mg/kg body weight orally daily, received a normal baseline food supplement until the conclusion of the experiment. The selected doses of lycopene were based on previous studies [10,11,57]. A typical basal diet with cholesterol (1%; *w*/*w*) was added to the third group (HCD). Just before the diets were given to the rats, the cholesterol batches were carefully combined with the basal diets [13]. The fourth and fifth groups received either oral lycopene (HCD-LYC; 50 mg/kg body weight) or atorvastatin (HCD-AV; 10 mg/kg body weight) every day in addition to a typical basal diet including cholesterol (1%; *w*/*w*).

### 4.2. Sampling and Tissue Preparation

Animal euthanasia after a 3 month intervention period was carried out following overnight fasting, during which time water was given as usual. Pentobarbital (300 mg/kg, i.p.) was administered in excess to kill the rats. Serum was collected after centrifuging at 3000× *g* for 15 min at 4 °C, and the obtained serum was immediately frozen at −80 °C for biochemical analysis. The kidneys and heart were taken out immediately. A 10 times volume of ice-cold 0.5 M potassium phosphate buffer (pH 7.4) was used to homogenize a part (100 mg) from the isolated kidney and heart tissues. Centrifugation at 3000× *g* (4 °C) for 10 min was used to separate the supernatant. For biochemical examination, the supernatants were kept at −80 °C. Prior to gene expression analysis, a portion of the kidney and heart tissues was taken and kept at −80 °C.

### 4.3. Glycemic Features and Serum Biochemical Parameters

The collected sera were evaluated for glucose by the enzymatic colorimetric test method GOD–PAP (Elabscience, Houston, TX, USA) as described by Trinder [58], whereas serum insulin levels were assessed using the ELISA method (MyBioSource, San Diego, CA, USA). Additionally, urea and creatinine were determined to evaluate the kidney function according to the manufacturer’s procedures (RANDOX Reagents; London, UK). Serum creatine kinase MB (CK; FineTest, Wuhan Fine Biotech Co., Ltd., Wuhan, China), lactate dehydrogenase (LDH; Sigma-Aldrich, St. Louis, MO, USA), and cardiac troponin T (cTnT) was determined by kit bought from MyBioSource (San Diego, CA, USA).

### 4.4. Lipid Profile and Atherogenic Index

Serum total cholesterol (TC) level of rats was measured using a commercial kit (Cat No: CH200; RANDOX Reagents, London, UK). Triacylglycerols (TG) in serum samples were estimated by a RANDOX kit (Cat No: TR210). High-density lipoprotein (HDL-c) in serum samples was calculated by HDL precipitant method using a commercially available Elabscience kit (Cat No: E-BC-K222-S; Houston, TX, USA). Furthermore, LDL-c was calculated by using the following formula (Friedewald formula [59]): LDL-c = TC − (HDL-c + TG/5). For the determination of the atherogenic index (AI), the method of Kazemi et al. [60] was used based on the following formula:$\;AI=\frac{\mathrm{TC}\;-\;\text{HDL-c}}{\text{HDL-c}}$.

### 4.5. Investigation of Cardiac and Renal Tissue Oxidative Stress Indicators

The thiobarbituric acid reaction method was used to measure the amount of malondialdehyde (MDA), a sign of lipid peroxidation [61], using a commercial kit (FineTest, Wuhan Fine Biotech Co., Ltd., Wuhan, China). Additionally, glutathione (GSH) content was measured using the Ellman method [62], and nitric oxide (NO) level was assessed using the Griess solution method [63], utilizing a colorimetric kit obtained from BioVision (Waltham, MA, USA).

### 4.6. Investigation of Antioxidant Enzyme Activity in Cardiac and Renal Tissues

The Nishikimi et al. method was used to assess the superoxide dismutase (SOD) activity in supernatants [64]. The Aebi [65] method was used to measure the catalase (CAT) activity. Based on the methods outlined by Mannervik [66] and Agergaard and Jensen [67], respectively, glutathione reductase (GR) and glutathione peroxidase (GPx) were estimated.

### 4.7. Identification of Pro-Inflammatory Cytokines in Cardiac and Renal Tissues

Tumor necrosis factor-alpha (TNF-α, Cat. No.: E-EL-R2856) and interleukin-6 (IL-6, Cat. No.: E-EL-R0015) levels were determined using commercial ELISA kits from Elabscience, Houston, TX, USA, in accordance with the manufacturer’s manual guidelines. In contrast, nuclear factor kappa B (NF-κB, Cat. No. CSB-E13148r) was quantified using an ELISA kit purchased from Cusabio in Wuhan, China, in accordance with the manufacturer’s instructions. Intercellular adhesion molecule 1 (ICAM-1) level was assessed by a commercial ELISA kit from Abcam, Cambridge, UK, in accordance with the manufacturer’s manual protocol.

### 4.8. Identifying Apoptotic Proteins in Cardiac and Renal Tissues

The quantities of the pro-apoptotic proteins (Bax, E-EL-R0098 and caspase-3, E-EL-R0160) and the anti-apoptotic protein (Bcl-2, E-EL-R0096) were measured using ELISA kits in accordance with the manufacturer’s manual protocol (Elabscience, Houston, TX, USA).

### 4.9. Analysis of Gene Expression

A TRIzol reagent (Invitrogen, Carlsbad, CA, USA) was used to extract total RNA from heart and kidney tissues in accordance with the manufacturer’s recommendations. Then, cDNA was created using reverse transcriptase from an aliquot of whole RNA. Power SYBR Green PCR Master Mix (Thermo Fisher Scientific, Waltham, MA, USA) was used for real-time PCR experiments, and an Applied Biosystems 7500 was used for analysis. As a reference gene, glyceraldehyde 3-phosphate dehydrogenase, also known as GAPDH, was used. The primer sequences are listed in Table 3.

### 4.10. Statistical Analysis

The mean and standard error of the mean are used to present data. To compare differences across various groups, a one-way analysis of variance was used along with Turkey’s honestly significant difference (HSD) post hoc analysis for multiple comparison tests. The threshold for statistical significance was set at *p* less than 0.05.

## 5. Conclusions

The results of this study reveal that lycopene, due to its anti-hyperlipidemic and anti-oxidative capabilities, protects the kidney and hearts of rats from experimental hypercholesterolemia. Atherosclerosis and atherogenic diet-mediated inflammation are also prevented by its anti-inflammatory impact, which also appears to be caused by reducing the activation of inflammatory markers, such as NF-κB, ICAM-1, and pro-inflammatory cytokines. Additionally, lycopene inhibits caspase-3 and Bax, two proteins that promote apoptosis, and enhances the antiapoptotic protein Bcl-2. However, further studies should be conducted to deeply investigate the mechanism behind those observations and to determine the appropriate dose and any adverse effects that may appear after lycopene usage in humans.

## Figures and Tables

**Table 1:** Effects of dietary cholesterol and lycopene on body weight and organ weight (absolute and relative) in rats over 12 weeks.

Experimental Groups	Control	LYC	HCD	HCD-LYC	HCD-AV
**Body weight at beginning (g)**	193.09 ± 9.06	192.67 ± 7.63	195.24 ± 8.43	194.60 ± 5.08	191.11 ± 5.92
**Body weight at end (g)**	273.27 ± 8.85	265.30 ± 10.07	384.84 ± 26.40 ^a^	313.52 ± 13.77 ^ab^	351.42 ± 15.70 ^ab^
**Body weight gain (g)**	80.17 ± 11.81	72.63 ± 16.33	189.60 ± 25.40 ^a^	118.92 ± 13.62 ^ab^	160.30 ± 18.96 ^ab^
**Absolute liver weight (g)**	8.13 ± 0.43	7.87 ± 0.60	10.56 ± 0.86 ^a^	9.50 ± 0.81 ^ab^	10.40 ± 0.65 ^a^
**Relative liver weight (g)**	2.97 ± 0.13	2.97 ± 0.17	2.75 ± 0.28 ^a^	3.03 ± 0.20 ^b^	2.96 ± 0.22 ^b^
**Absolute kidney weight (g)**	1.73 ± 0.12	1.74 ± 0.14	1.92 ± 0.18 ^a^	1.83 ± 0.14	1.93 ± 0.12 ^a^
**Relative kidney weight (g)**	0.63 ± 0.06	0.66 ± 0.07	0.50 ± 0.07 ^a^	0.58 ± 0.05 ^b^	0.55 ± 0.06 ^a^
**Absolute heart weight (g)**	1.42 ± 0.06	1.43 ± 0.05	1.86 ± 0.12 ^a^	1.58 ± 0.06 ^b^	1.61 ± 0.09 ^b^
**Relative heart weight (g)**	0.52 ± 0.03	0.54 ± 0.03	0.49 ± 0.05	0.50 ± 0.03	0.46 ± 0.04 ^a^

Letters a and b indicate statistically significant differences between control and HCD groups, respectively, at *p* < 0.05.

**Table 2:** Effects of dietary cholesterol and lycopene on serum glycemic features, lipid profile, and kidney function parameters in rats over 12 weeks.

Experimental Groups	Control	LYC	HCD	HCD-LYC	HCD-AV
**Total cholesterol (mg/dL)**	86.58 ± 9.52	88.52 ± 11.79	253.69 ± 26.67	135.46 ± 19.15	115.23 ± 20.74
**Triglyceride (mg/dL)**	104.63 ± 15.61	106.01 ± 23.76	356.76 ± 57.36 ^a^	186.38 ± 39.17 ^ab^	162.54 ± 12.17 ^ab^
**LDL-c (mg/dL)**	14.71 ± 12.44	16.90 ± 9.00	154.61 ± 25.14 ^a^	48.98 ± 22.41 ^ab^	40.98 ± 25.71 ^ab^
**vLDL-c (mg/dL)**	20.93 ± 3.12	21.20 ± 4.75	71.35 ± 11.47 ^a^	37.28 ± 7.83 ^ab^	32.51 ± 2.45 ^ab^
**HDL-c (mg/dL)**	50.95 ± 8.61	50.41 ± 7.07	27.73 ± 3.74 ^a^	49.20 ± 6.20 ^b^	41.74 ± 6.59 ^b^
**Athrogenic index**	0.75 ± 0.44	0.78 ± 0.29	8.24 ± 1.23 ^a^	1.77 ± 0.39 ^ab^	1.86 ± 0.83 ^ab^
**Glucose (mg/dL)**	96.07 ± 12.54	100.72 ± 10.11	167.76 ± 25.78 ^a^	114.66 ± 19.18 ^b^	150.18 ± 15.99 ^a^
**Insulin (ng/mL)**	5.62 ± 0.93	5.72 ± 0.98	7.79 ± 1.78 ^a^	5.96 ± 1.08 ^b^	6.64 ± 0.91 ^b^
**Urea (mg/dL)**	48.52 ± 8.90	43.75 ± 6.88	132.85 ± 14.48	76.50 ± 10.53 ^ab^	106.68 ± 17.62 ^ab^
**Creatinine (mg/dL)**	0.54 ± 0.04	0.52 ± 0.07	1.08 ± 0.19 ^a^	066. ±0.07 ^b^	0.79 ± 0.07 ^ab^

Letters a and b indicate statistically significant differences between control and HCD groups, respectively, at *p* < 0.05.

**Table 3:** Primer sequences of genes analyzed in real-time PCR.

Name	Accession Number	Sense (5′→3′)	Antisense (5′→3′)
GAPDH	NM_017008.4	CTCTCTGCTCCTCCCTGTTC	TACGGCCAAATCCGTTCACA
PPAR-γ	NM_001145366.1	AGTAGCCTGGGCTGCTTTTAT	GATCACCAGCAGAGGTCCAG
PGC-1α	NM_031347.1	CATGTGCAGCCAAGACTCTG	GTGAGGACCGCTAGCAAGTT
PON-1	NM_032077.1	CAGGACATGGCGAAACTGCT	TCTAAGTCTTCAGCACCCGC

The abbreviations of the genes are as follows: GAPDH, glyceraldehyde-3-phosphate dehydrogenase; PPAR-γ, peroxisome proliferator-activated receptor gamma; PGC-1α, PPARG coactivator 1 alpha; PON-1, paraoxonase 1.

## Data Availability

All relevant data are within the paper.
